# Increased temperature has no consequence for behavioral manipulation despite effects on both partners in the interaction between a crustacean host and a manipulative parasite

**DOI:** 10.1038/s41598-020-68577-z

**Published:** 2020-07-15

**Authors:** Sophie Labaude, Frank Cézilly, Lila De Marco, Thierry Rigaud

**Affiliations:** 1 Université de Bourgogne Franche-Comté, UMR CNRS 6282 Biogéosciences, Dijon, France; 2 Present Address: Laboratoire “Génétique Evolutive Expérimentale”, Institut de Biologie de L’Ecole Normale Supérieure (IBENS), Paris, France

**Keywords:** Behavioural ecology, Climate-change ecology, Freshwater ecology, Parasitology

## Abstract

Parasites alter many traits of their hosts. In particular, parasites known as “manipulative” may increase their probability of transmission by inducing phenotypic alterations in their intermediate hosts. Although parasitic-induced alterations can modify species’ ecological roles, the proximate factors modulating this phenomenon remain poorly known. As temperature is known to affect host–parasite associations, understanding its precise impact has become a major challenge in a context of global warming. Gammarids are ecologically important freshwater crustaceans and serve as intermediate hosts for several acanthocephalan species. These parasites induce multiple effects on gammarids, including alterations of their behavior, ultimately leading to modifications in their functional role. Here, experimental infections were used to assess the effect of two temperatures on several traits of the association between *Gammarus pulex* and its acanthocephalan parasite *Pomphorhynchus laevis*. Elevated temperature affected hosts and parasites in multiple ways (decreased host survival, increased gammarids activity, faster parasites development and proboscis eversion). However, behavioral manipulation was unaffected by temperature. These results suggest that predicted change in temperature may have little consequences on the trophic transmission of parasites through changes in manipulation, although it may modify it through increased infection success and faster parasites development.

## Introduction

Abiotic conditions can strongly influence interspecific interactions and, ultimately, the dynamics of ecological communities. In direct connection with climate change, the understanding of such effect has become a major challenge in recent years^1–3^. Abiotic conditions, including temperature, can, for instance, affect predator–prey interactions and food-web dynamics^4–6^ or competition between species^7–11^. They can also directly or indirectly affect host–parasite interactions^12–14^, with cascading effects for trophic interactions and ecosystem stability^15–17^. This is all the more relevant in the case of parasite species that infest and modify the phenotype of host species known as ‘ecosystem engineers’, i.e. species that can affect the physical properties of ecosystems^18–20^.

For instance, some recent evidence suggests that changes in temperature can modulate the influence of parasitic infection on both the functional role and the coexistence of crustacean amphipod species^17,21–23^. Crustacean amphipods are widespread throughout a large range of freshwater habitats^24,25^, in which they play a key ecological role. They represent an important food resource for many species^26,27^ and are themselves a major predator^24,28^, capable of modulating the composition of freshwater macroinvertebrates communities^28,29^. Some species can also directly influence water quality and the recycling of organic matter through their shredder role on dead leaves^25,30–32^. Crustacean amphipods serve as intermediate host for a large variety of helminths with complex life-cycles^33^. In particular, the association between amphipods and acanthocephalan parasites (thorny-headed worms) has received considerable attention^34,35^. Acanthocephalans can affect the phenotype of their amphipod hosts in many different ways, through altering their behavior^36–38^, their feeding and predatory activity^23,39,40^, their immune system^41^, their energetic reserves^42^, their fecundity^43,44^, or their metabolic rate^45,46^, ultimately leading to modifications in the role of amphipods within ecosystems. In particular, amphipods infected with acanthocephalans tend to be more vulnerable to predation, especially by final hosts of the parasites^47–51^.

As for other ectotherm species^52^, temperature has been shown to affect amphipods in several ways, influencing for instance their metabolism^53–55^, growth^56^, or activity^54^. Similarly, most parasites are also affected by temperature in diverse ways^57^. For instance, temperature affects both the development time of acanthocephalan parasites in their intermediate hosts^58,59^ and their prevalence and abundance in their definitive fish hosts^60^. This is reflected in field studies having reported seasonal effects in the prevalence and intensity of infection with acanthocephalans in both intermediate and definitive hosts^61–65^. So far, however, only a limited number of studies have provided evidence for a joint effect of temperature and infection with acanthocephalan parasites on the ecological role of their intermediate host species. For instance, Labaude et al.^23^ showed that infection with acanthocephalan parasites and temperature have additive effects on the shredding role of crustacean amphipods, whereas Guinnee and Moore^66^ reported an interaction between temperature and infection with acanthocephalans on the fecundity of insect hosts.

The influence of increased temperature on the phenotypic alterations induced by acanthocephalans on their intermediate hosts has received little attention despite their potential consequences at the ecosystem level. Although the exposition of naturally-infected isopods to different conditions of light and temperature resulted in changes in behavior, no differences were found in the extent of manipulation^67^. However, isopods used in this study were naturally-infected, such that conditions during parasite development were not controlled. In addition, the effects of temperature and light were not investigated separately, although previous studies showed that the manipulation of gammarids by acanthocephalans depends to some extent on light properties^68,69^. Temperature was shown to affect significantly the alteration of phototaxis induced by natural infections of the acanthocephalan *Pomphorhynchus tereticollis* in *Gammarus pulex*^22^. However, there was no evidence for an effect of temperature on the use of refuge by infected vs. uninfected gammarids, thus suggesting that temperature may have different effects on different dimensions of manipulation^22^. Additional studies in which both temperature and parasite infection are controlled experimentally would thus help to better understand how temperature affects host manipulation by acanthocephalan parasites.

Following experimental infections, we investigated the effect of two temperatures on the magnitude of phenotypic alterations induced by *P. laevis* on *G. pulex*, including the intensity and timing of manipulation. After parasites reached the cystacanth stage, at which they become infective for the definitive host, the use of refuge by gammarids (a behavior known to be directly involved in parasite trophic transmission^47,49^) was measured. Other parameters of both hosts and parasites (i.e. host survival and activity level, rapidity of parasites’ proboscis eversion), as well as infection parameters (i.e. infection success, parasite load, speed of parasites development), were also recorded.

## Methods

### Sampling

Uninfected *G. pulex* gammarids were collected in a small tributary of the Suzon River (eastern France, 47° 24′ 12.6″ N, 4° 52′ 58.2″ E) in October. Gammarids from this population have been widely used in previous studies for experimental infections, such that the system is now well characterized^45,70–74^. Previous studies did not show any effect of the sex of gammarids on the extent of behavioral modifications^37,73,75,76^. However, failure in parasite infection was observed in female gammarids more often than in males^73^. Therefore, only males were used in this study. Before experimental infections, gammarids were maintained in the laboratory at 15 °C and under a 12:12 light:dark cycle.

The Vouge River (eastern France, 47° 9′ 34.36″ N 5° 9′ 2.50″ E) was chosen to collect parasites from naturally infected chubs (*Leuciscus cephalus*) in October, as this population of parasites has previously been shown to be highly infective to gammarids in the lab^74^. We extracted acanthocephalan eggs from adult parasites sampled in the intestines of the fish. Because both *P. laevis* and *P. tereticollis* parasites can be found in fish from this river and cannot be distinguished visually, the species of adult parasites was determined using genetic analyses with the method described in Franceschi et al.^73^. Only eggs from *P. laevis* were used for experimental infections.

A second sampling of gammarids and fish was conducted in November and December for control experiments (see below).

### Experimental infections and treatments

Gammarids were experimentally exposed to *P. laevis* eggs following the procedure detailed in Franceschi et al.^73^. Pairs of gammarids that were previously starved for 24 h in glass dishes were exposed for 48 h to 200 parasite eggs (100 eggs per gammarid being a good compromise between a high infection success and a low rate of multiple infections^73^). Gammarids were then placed in individual glass dishes, and randomly distributed among the different treatments. Control individuals were maintained under the same conditions, without parasite eggs.

**Figure 1:**
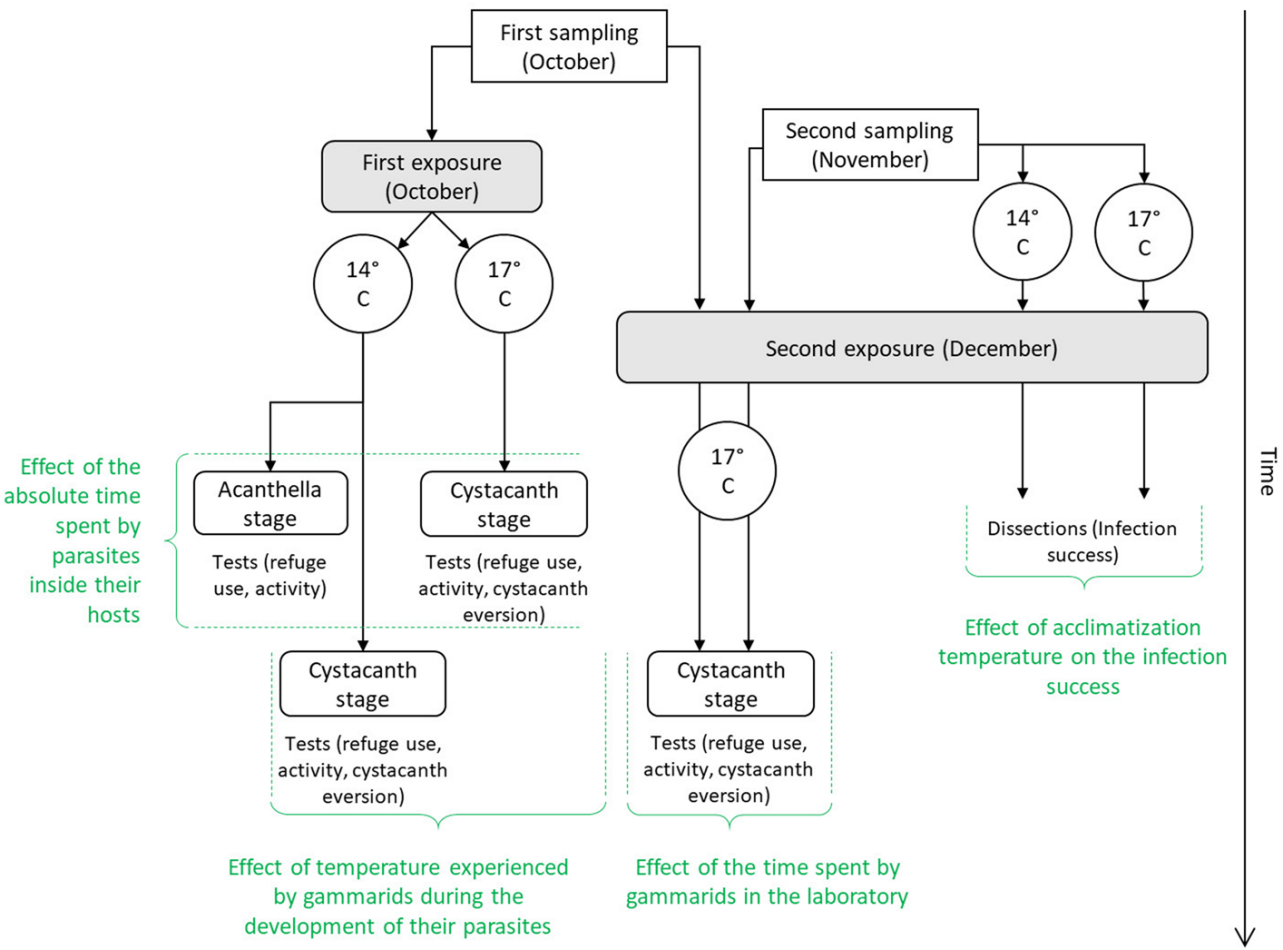
Overview of the protocol used in this study. Control individuals were maintained in the same conditions as exposed individuals and were simultaneously tested. Captions in green indicate which parts of the experiment were used for each test.

To investigate the effect of temperature during the development of parasites, 1,200 gammarids were exposed to parasite eggs, and 420 individuals were maintained as control. Immediately following the exposure, gammarids were allocated to two temperature treatments: normal temperature of 14 °C—which is naturally experienced by gammarids in the field during summer—and increased temperature—corresponding to an addition of 3 °C consistent with scenarios of climate change^77–79^ (Fig. 1).

Because the development time of parasites is highly dependent on temperature^58,59^, parasites were expected to reach the cystacanth stage later at 14 °C compared to 17 °C. Consequently, gammarids at 14 °C would spend more time in the laboratory before being tested. Therefore, a second experimental infection was conducted to control for the effect of time spent by gammarids in the laboratory (Fig. 1). Additional gammarids from the first sampling (October) were maintained for 40 days at 17 °C before being exposed to parasite eggs sampled in December (n = 650), or kept as controls (n = 120). In parallel, 90 freshly sampled gammarids (second sampling, November) were experimentally infected with the same parasite eggs, and 30 individuals were used as controls. Following the second infection, all gammarids were maintained in individual glass dishes at 17 °C (see Fig. 1).

All individuals were maintained under a 12:12 light:dark cycle, and were fed ad libitum with conditioned elm leaves, plus one additional frozen chironomid larvae once every 2 weeks, ensuring improved survival of gammarids^45^. Water was changed once every 2 weeks, using an oxygenated mix of water from the Suzon River and dechlorinated, UV-treated tap water.

### Monitoring

All gammarids were inspected on a daily basis to record the death of any individual. Individuals that were exposed to parasites were dissected immediately after their death to determine their infection status (number of parasites and their development stage). In addition, 150 control and 150 gammarids exposed to parasite eggs were randomly selected at each temperature at the beginning of the experiment. Individuals found dead among them were then measured from body height at the level of the fourth coxal plate^80^ using a microscope and Lucia G 4.81 software (Prague, Czech Republic).

Following detection of the first parasites at the advanced acanthella stage (> 1,000 µm) upon dissection, all individuals were checked daily under a binocular microscope to monitor the exact date of the switch between the acanthella stage (ovoid shape, translucent orange color) and the cystacanth stage (spherical and more pronounced opaque color^81^). Behavioral tests were then conducted (see below).

At the end of the experiments, we measured and dissected all gammarids that were used in behavioral tests, including control individuals. Although the presence of *P. laevis* has not been recorded in the Suzon River, gammarids from this population can be naturally infected with other acanthocephalan parasite species (*Echinorhynchus truttae* and *Polymorphus minutus*), as well as other macro-parasites such as *Cyathocephalus truncatus* (Cestoda). Such infected individuals were removed from the data set.

### Measurement of refuge use

The use of refuges by gammarids was recorded on three consecutive occasions (hereafter referred as “rounds”) for all infected individuals: 1 day, 8 days, and 16 days after the cystacanth stage was detected. We tested control individuals in a similar fashion in parallel. Gammarids were placed in individual boxes (10.5 × 16 cm) filled with 250 ml of water, containing a refuge that consisted of a saucer terracotta pot (8.5 cm of diameter) cut in half, with a 1 cm hole in the convex part (see Dianne et al.^82^). After 10 min of acclimatization following the introduction of gammarids, we recorded the position of each individual every 2 min for 60 min. For each observation, a score of zero was given to individuals that were outside of the refuge, and one for individuals inside it. Summed scores at the end of each round thus ranged from 0 (always outside the refuge) to 30 (always inside) for each individual.

As expected, the development of parasites in gammarids maintained at 14 °C was longer than that of gammarids maintained at 17 °C. We therefore tested whether the timing and intensity of manipulation depended on the absolute time that parasites spent into their hosts rather than on their development stage, as well as the effect of the time spent by gammarids in the laboratory (both tests and their results are detailed in the Supplementary material 1).

All gammarids were tested at their acclimatization temperature. In order to be able to perform tests at different temperatures in one single room, test boxes were placed in water baths, with surrounding water constantly recirculated through a temperature control device (Tank TK-1000 Chiller, Teco, Ravenna, Italy; see Labaude et al.^23^).

### Measurement of activity

The activity level of individual gammarids was tested 3 days after the second round of refuge use tests (i.e. 11 days after the detection of the cystacanth stage for infected individuals). The apparatus consisted of a 10-cm diameter glass dish containing a smaller dish (6-cm diameter) preventing the gammarid to go in the centre of the larger glass dish, thus forming a 2-cm wide annulus. To limit vertical movements of the individual, the apparatus was filled with only 1 cm of water. Lines were traced under the glass dish, intersecting in their centre, thus dividing the annulus into eight equally large zones. After 5 min of acclimatization following the introduction of the gammarid in the device, the behavior of the individual was video-recorded from above for 5 min. The activity level of the individual was expressed as the number of lines that it crossed during 5 min.

### Rapidity of proboscis eversion

The rapidity of cystacanth parasites to evert their proboscis was measured in old cystacanths (between 20 and 30 days after their detection) extracted from gammarids previously tested for their behavior. The eversion of the proboscis, which allows parasites to attach to the intestine wall of their fish host, is a crucial step for a parasite to complete its life cycle and incurs a physiological cost as an active process^83,84^. Thus, latency to evert the proboscis can be used as a proxy to estimate parasite stamina^85^. Immediately following the dissection of their hosts, each cystacanth parasite was carefully placed in a 96-well microplate. The eversion of cystacanth proboscis is known to occur in reaction to a component of fish bile^86^. Therefore, 30 µl of bile extracted several months earlier from European chubs, *Squalius cephalus*, frozen for conservation since then, and diluted 30 times with water, were added to each microplate well. The microplate was immediately covered with aluminium foil to limit evaporation, and with an opaque box to ensure darkness (see Perrot-Minnot et al.^85^). Each microplate, containing no more than 20 cystacanths, was then checked every 5 min under a dissecting microscope, with reduced light, and quickly replaced in the dark. We recorded the time needed for each parasite to start to evert their proboscis. The temperature was kept at 15 °C for all measurements of proboscis eversion, regardless of the treatment group.

### Effect of acclimatization temperature on prevalence

In the experiments previously described, all gammarids were exposed to parasite eggs at the same temperature (15 °C), before being maintained at 14 °C or 17 °C. Therefore, we could not draw any conclusion about the effect of temperature on the success of parasite infection. Indeed, although temperature might affect the success of establishment of parasites, it is also known to modify the consumption rate of gammarids^23,87^, thus possibly affecting their probability of consuming parasite eggs and getting infected at different temperatures. Therefore, to measure the effect of temperature on the success of parasite infection, 100 gammarids from the second sampling (November) were acclimatized for 3 weeks at each of the two temperatures (14 °C or 17 °C), in individual glass dishes. We then exposed gammarids to parasite eggs collected in December, following the protocol described above, at their acclimatization temperature (Fig. 1). Conditions were kept similar during parasite development. All gammarids were dissected immediately after their death or at the end of the experiment to determine their infection status.

### Statistical analyses

The survival of gammarids was analyzed using Cox regressions. First, the effects of infection status (control vs infected) and temperature were analyzed. A second Cox regression was performed using only infected individuals to investigate the effect of parasite load (one, two, or more than two parasites per gammarid) and temperature. Once parasites were large enough to be detected upon dissection, individuals that were exposed to parasite eggs in which no parasite developed were removed from the analyses.

We used nominal logistic regressions to investigate the success of infection (i.e. the proportion of gammarids harboring at least one parasite among those exposed to the infection), and Mann–Whitney U tests to compare the parasite load between the groups as well as the time needed for parasites to reach the cystacanth stage.

Scores for refuge use were analyzed as repeated measures using the ‘nparLD’ R software package. This function is suitable for nonparametric analyses of right-censured longitudinal data, allowing the decrease in sample size along time, due to individuals’ death^88^. First, the effects of temperature (14 °C vs 17 °C), infection status (control vs infected) and their interaction were investigated along time (rounds of measurements: 1 day, 8 days and 16 days after parasites reached the cystacanth stage). A second analysis was conducted on infected individuals only, with temperature, parasite load, their interaction, and rounds as factors. For each analysis, ‘ANOVA-type statistics’ were performed, followed by post-hoc pair-comparisons when suitable (see Noguchi et al.^88^ for details). Because parasites developed faster at high temperature, the size of gammarids that were killed following behavior tests was measured later at 14 °C compared to 17 °C. We therefore suspected that gammarids may have had more time to make an additional molt at 14 °C than at 17 °C, and thus could be larger. An ANOVA confirmed that the size of gammarids was globally larger at 14 °C (F_399,1_ = 5.84, *P* = 0.016). However, within each temperature, we found no general correlation between refuge scores and the size of gammarids, neither at 14 °C (Spearman correlation, rho = 0.064, *P* = 0.056) or at 17 °C (rho = 0.05, *P* = 0.21). This suggests that size does not influence this behavior. Therefore, to avoid confusion between size and temperature in our analyses, the size of individuals was not considered in between-temperatures analyses of refuge score.

The activity level of gammarids was investigated using a linear model (ANOVA), followed by Tukey post-hoc tests.

Latency time to evert the proboscis was investigated using a Generalized Linear Model with a Poisson distribution corrected for over-dispersion. We tested for the effects of temperature, parasite load, the time needed for each parasite to reach the cystacanth stage, and their interactions. Spearman correlations were used to test if there was a link between how fast parasites were everting their proboscis and their ability to manipulate the behavior of their hosts (scores of refuge use and activity level) at each temperature.

Statistical analyses were performed using JMP version 10.0.0 software (SAS Institute, Cary, NC, USA) and R version 3.1.1 software (R Foundation for Statistical Computing, Vienna, Austria). For each analysis described above, all factors and their second order interactions were first entered in the models. Non-significant factors or interactions were then removed.

### Ethical statement

The animals were captured, transported and handled in accordance with article L.436-9 of the Environment Code, with the authorization of the Prefectural Decree of Côte-D'or no. 77 from the 11/02/2019. The fish for parasites samplings were killed after being anesthetized, in accordance with French regulations on the ethics of animal testing (decree 2013-118).

## Results

### Effect of temperature on survival

**Figure 2:**
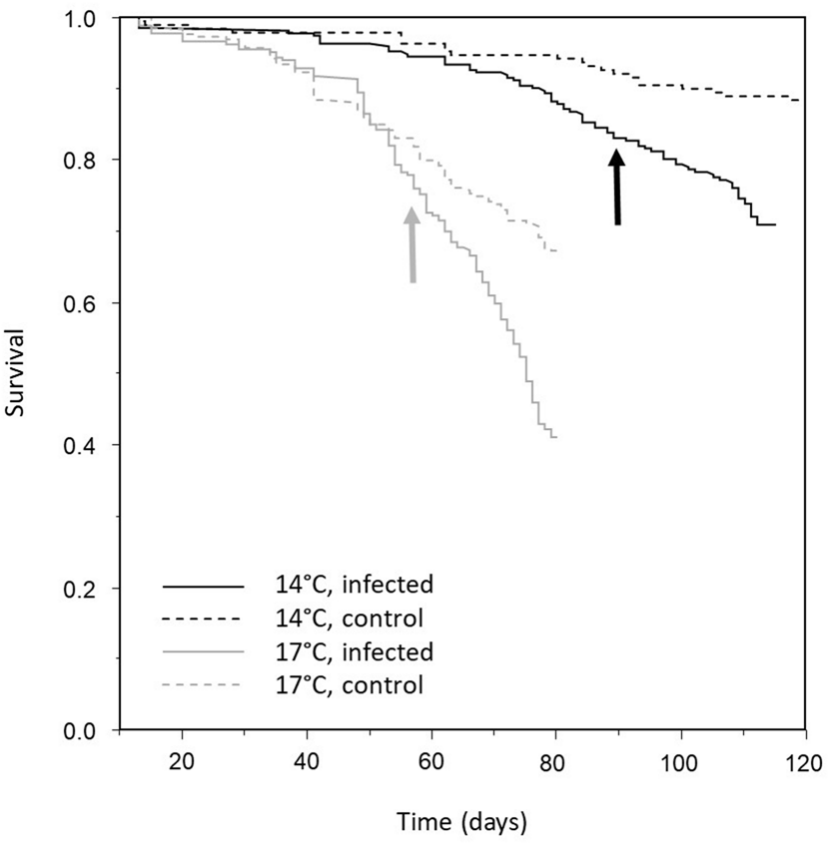
Survival of gammarids according to infection status (control or infected by *P. laevis* parasites) and temperature (14 °C or 17 °C). Time 0 was considered as the day on which gammarids were exposed to parasite eggs. Arrows indicate the average day of switching of parasites between acanthella and cystacanth stages at each temperature.

Cox regression on gammarids from the first exposure (Global model: Likelihood-Ratioχ^2^ = 208.97, df = 2, *P* < 0.0001) showed that survival was significantly higher at 14 °C compared to 17 °C (LR-*χ*^2^ = 188.56, df = 1, *P* < 0.0001, Fig. 2). Control individuals also survived significantly better than infected individuals (LR-*χ*^2^ = 34.88, df = 1, *P* < 0.0001, Fig. 2). The virulence of parasites did not differ between temperatures, as indicated by the absence of significant interaction between temperature and infection. When considering infected individuals only, no effect of parasite load on the survival of gammarids was found, and only temperature remained in the model (LR-*χ*^2^ = 144.09, df = 1, *P* < 0.0001).

### Effect of temperature on infection parameters

Overall, 53.8% of individuals that were exposed to parasite eggs were successfully infected. When gammarids were exposed to eggs at the same temperature before being distributed among the two temperature treatments, there was no difference between the two temperatures in both the success of infection (LR-*χ*^2^ = 0.003, df = 1, *P* = 0.96) and the parasite load (mean ± standard deviation = 1.67 ± 1.04 parasites per gammarid, Mann–Whitney U test: *Z* = 1.06, *P* = 0.29). Development time of parasites (from exposure to cystacanth stage) was however significantly longer at 14 °C (mean ± standard deviation = 89.95 ± 2.49 days) than at 17 °C (57.27 ± 1.39 days; Mann–Whitney U test, *Z* = − 14.69, *P* < 0.0001; Fig. 2).

When gammarids were acclimatized for 3 weeks at the two temperatures before exposure to parasite eggs, infection success tended to be higher at 17 °C (84.9%; n = 86) than at 14 °C (74%; n = 77), although the difference was not significant (LR-*χ*^2^ = 2.97, df = 1, *P* = 0.08). More parasites developed in gammarids at 17 °C (mean ± standard deviation = 4.58 ± 2.76 parasites per gammarid) compared to 14 °C (mean ± standard deviation = 3.86 ± 2.55 parasites per gammarid), although this difference was only very close to significant (Mann–Whitney U test, *Z* = − 1.96, *P* = 0.05).

### Effect of temperature on the behavior of gammarids

**Figure 3:**
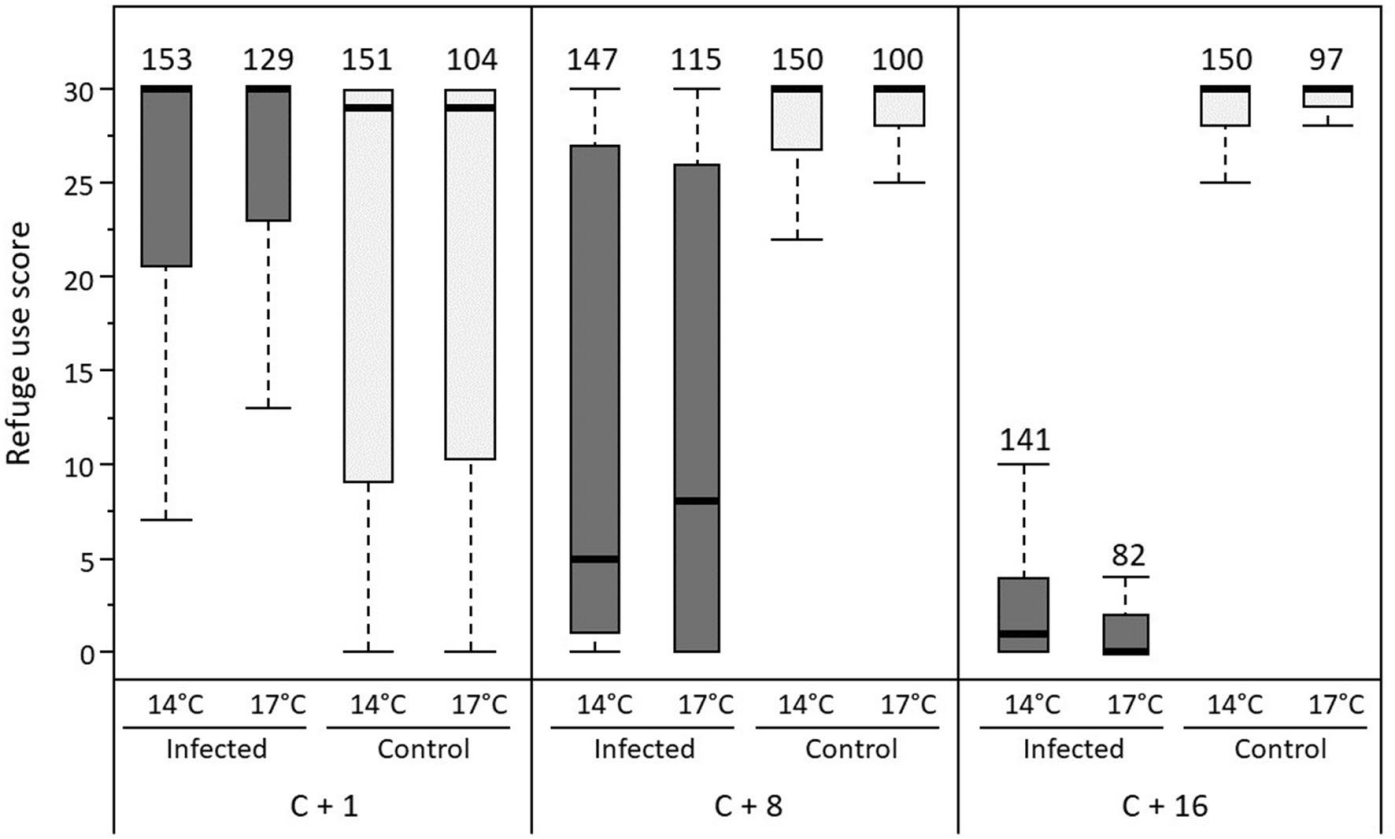
Scores of refuge use by gammarids (high scores refer to more time inside the refuge) according to infection status (infected or control) and temperature (14 °C or 17 °C), measured during three rounds: 1 day (C + 1), 8 days (C + 8) and 16 days (C + 16) after detection of cystacanth stages. Scores range from 0 (individuals always outside the refuge) to 30 (individuals always inside the refuge). Thick lines represent the medians, boxes represent the upper and lower quartiles, and dotted lines represent the upper and lower deciles. Sample sizes are given above each plot.

ANOVA-type results from the nparLD model showed that temperature did not influence the use of refuges by gammarids (Statistic = 0.63, df = 1, *P* = 0.43; Fig. 3), justifying the removal of temperature from the model. The remaining model showed that infection status, time (behavioral rounds), and the interaction between these two factors significantly influenced refuge use (Table 1). The use of refuges decreased with time for infected individuals, whereas it increased for control ones (Fig. 3).

**Table 1:** Results of the model from the nparLD R package, testing for the effects of infection status (infected with *P. laevis* cystacanths or control) and rounds of measurement on the scores of refuge use by *G. pulex* individuals.

Factor	Statistic	df	*P*
Status	323.51	1	< 0.0001
Round	124.56	1.96	< 0.0001
Status × round	393.86	1.96	< 0.0001

**Figure 4:**
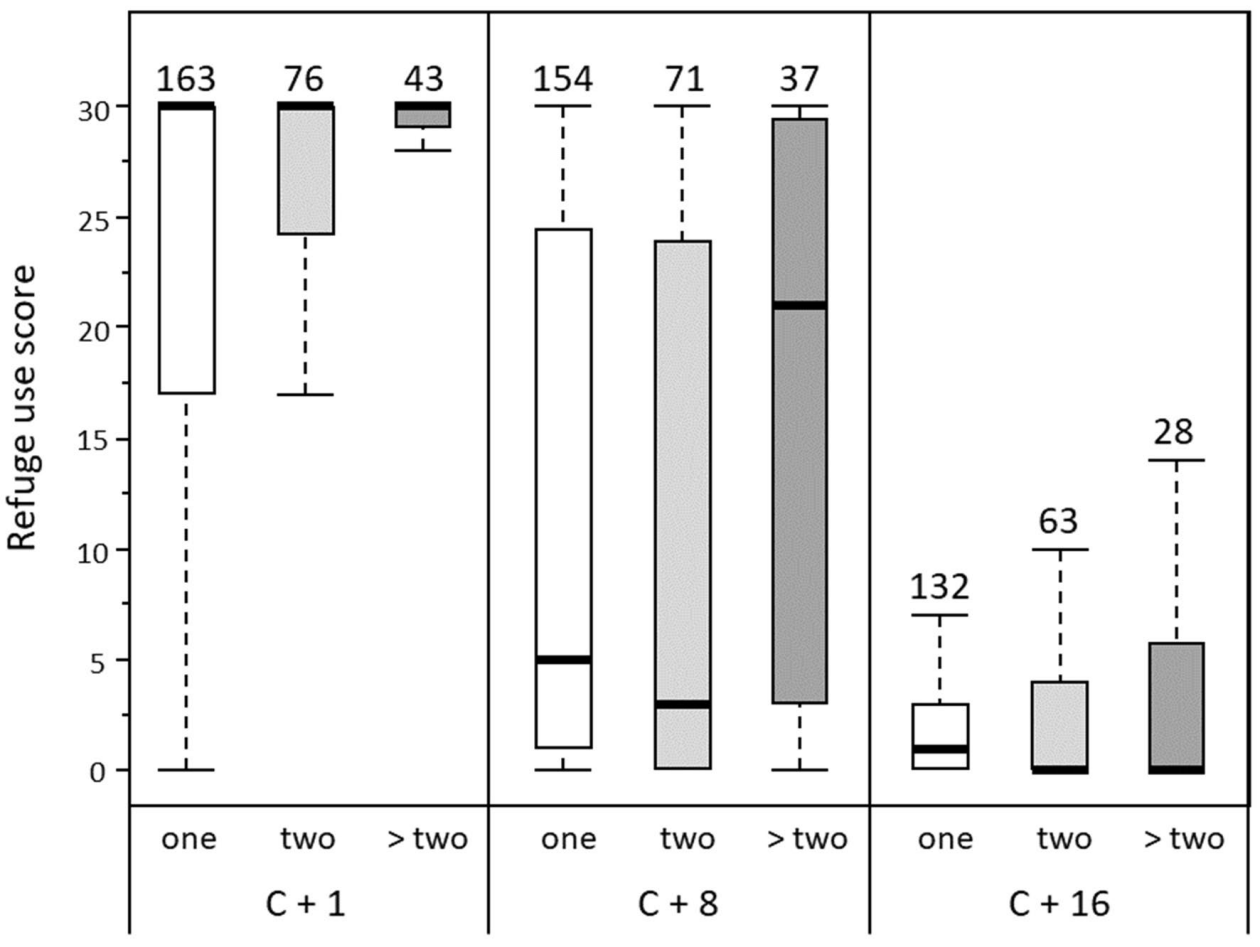
Scores of refuge use by infected gammarids (high scores refer to more time inside the refuge) according to parasite load (one, two or more than two parasites per gammarid), measured during three rounds: 1 day (C + 1), 8 days (C + 8) and 16 days (C + 16) after the detection of cystacanth stages. Scores range from 0 (individuals always outside the refuge) to 30 (individuals always inside the refuge). Thick lines represent the medians, boxes represent the upper and lower quartiles, and dotted lines represent the upper and lower deciles. Sample sizes are given above each plot.

When considering only infected individuals to investigate the effect of parasite load (one, two or more than two parasites), temperature had again no effect on the use of refuges (Statistic = 1.19, df = 1, *P* = 0.27) and was thus removed from the analysis. Although the remaining model confirmed that infected gammarids decreased their use of refuges over time, we found that parasite load and its interaction with time also significantly affected the use of refuges by gammarids (Table 2). Paired comparisons showed that, overall, refuge use was lower in gammarids infected with one or two parasites compared to gammarids infected with more than two parasites (Fig. 4, pair-comparisons 2 and 3 in Table 2). In addition, while all groups reached similar scores on the third round, their dynamics was different across time (this difference being significant only between gammarids harboring one and those harboring more than two parasites). In particular, the switch from refuge use to refuge avoidance happened later in gammarids with more than two worms (Fig. 4, pair-comparison 2 in Table 2).

**Table 2:** Results of the model from the nparLD R package, testing for the effects of parasite load (one, two or more than two *P. laevis* cystacanths per gammarid) and rounds of measurement on the scores of refuge use by *G. pulex* individuals.

Factor	Statistic	df	*P*
**ANOVA test**			
Parasite load	3.51	1.82	0.034
Round	310.64	1.91	< 0.0001
Parasite load × round	2.93	3.17	0.030
**Paired comparisons**			
***(1) Gammarids infected with one and two parasites***			
Parasite load	0.026	1	0.87
Round	313.45	1.93	< 0.0001
Parasite load × round	2.76	1.93	0.065
***(2) Gammarids infected with one and more than two parasites***			
Parasite load	4.93	1	0.026
Round	183.76	1.86	< 0.0001
Parasite load x round	3.49	1.86	0.033
***(3) Gammarids infected with two and more than two parasites***			
Parasite load	4.39	1	0.036
Round	178.42	1.87	< 0.0001
Parasite load × round	2.55	1.87	0.082

Only infected individuals were considered in the analysis.

**Figure 5:**
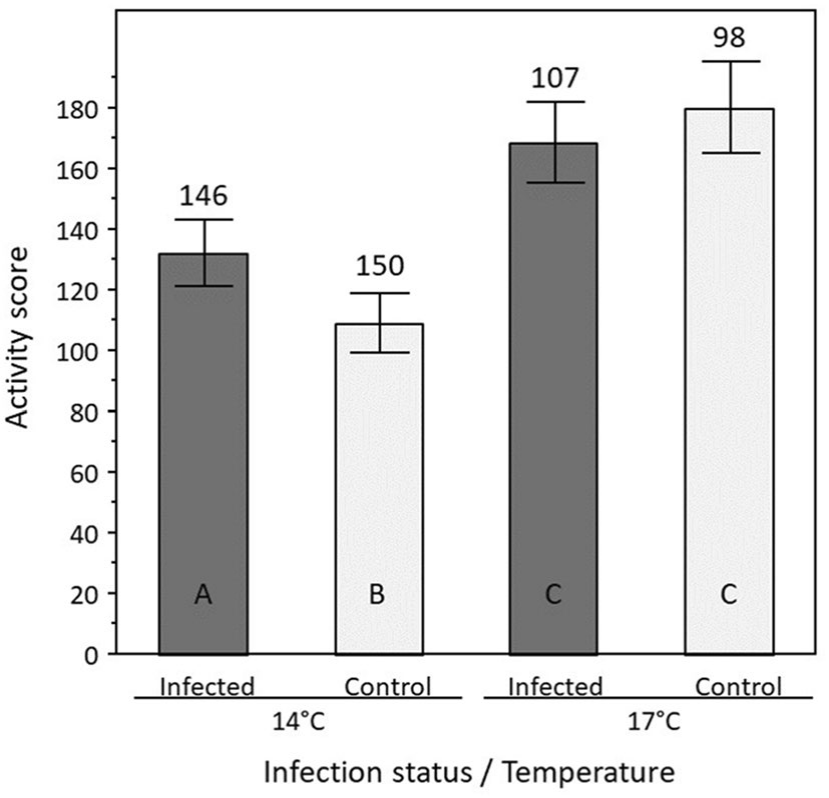
Activity level of gammarids according to infection status (infected by *P. laevis* cystacanths or control) and temperature (14 °C and 17 °C). Level of activity is given by a score corresponding to the number of zones entered during 5 min in an annulus-shaped arena. Mean values and 95% confidence intervals are indicated. Sample sizes are given above each bar. Significant differences are indicated by different letters (Tukey’s HSD post hoc tests; *P* < 0.05).

Temperature significantly affected the level of activity of gammarids (ANOVA *F*_3,500_ = 29.05, *P* < 0.0001). Individuals were significantly more active at 17 °C compared to 14 °C (*F*_1,500_ = 77.14, *P* < 0.0001, Fig. 5). Although infection status alone was not significant (*F*_1,500_ = 0.89, *P* = 0.35), its interaction with temperature also influenced gammarids activity level (*F*_1,500_ = 7.99, *P* = 0.005). Tukey’s HSD post hoc tests showed that the activity of infected individuals was significantly higher than that of control individuals at 14 °C (*P* = 0.0019), but not at 17 °C (*P* = 0.26; Fig. 5).

### Effect of temperature on parasites stamina: latency time to evert the proboscis

The Generalized Linear Model (*χ*^2^ = 6.13, df = 2, *P* = 0.047) showed that proboscis eversion, considered as a proxy to estimate parasite stamina^85^, was slightly but significantly faster for parasites that developed in hosts maintained at 17 °C (median and interquartile range = 40 [35; 45] min) compared to hosts maintained at 14 °C (median and interquartile range = 45 [40; 50] min, LR-*χ*^2^ = 5.57, df = 1, *P* = 0.018). In addition, there was a negative relation between the time taken by parasites to reach the cystacanth stage and the latency time to evert their proboscis, with eversion starting sooner for parasites that took more time to develop (LR-*χ*^2^ = 4.97, df = 1, *P* = 0.026).

Spearman correlations showed that proboscis eversion was faster in parasites inducing a less intense manipulation in the third round of the refuge use test, although the correlation was significant only at 17 °C (Table 3). No other behavior of gammarids was correlated with the rapidity of proboscis eversion of their parasites (Table 3).

**Table 3:** Spearman correlations between the latency time for *P. laevis* cystacanth parasites to start the eversion of their proboscis and the behavioral scores of their hosts.

Group	Factor	rho	n (parasites)	*P*
14 °C	Activity	0.005	199	0.95
	Refuge, first round	0.02	199	0.75
	Refuge, second round	− 0.04	199	0.59
	Refuge, third round	− 0.08	199	0.29
17 °C	Activity	0.17	94	0.10
	Refuge, first round	− 0.07	94	0.51
	Refuge, second round	− 0.08	94	0.43
	Refuge, third round	− 0.26	94	**0.01**

Significant value (after Bonferroni correction, *α* = 0.05) is highlighted in bold.

## Discussion

Higher temperature resulted in decreased survival and increased activity in gammarids, as well as in faster cystacanth development and quicker proboscis eversion in parasites. An increased parasite load with increased temperature during exposition of amphipod hosts to parasite eggs was also observed. Despite all these effects, neither the timing nor the intensity of manipulation (as assessed by refuge use) was affected by temperature once the parasites had reached the cystacanth stage.

As expected based on previous studies^58,59^, the time of development of parasites was much longer (57%) at 14 °C compared to 17 °C, with a remarkable synchrony within temperatures, all parasites switching between the acanthella and cystacanth stages within a few days at each temperature. Because of this difference, gammarids at 14 °C spent more time in laboratory conditions than those at 17 °C before being tested for behavior. In addition, we conducted behavioral tests while parasites were at the same development stage, but not at the same age. However, control tests allowed us to discard these two potentially confounding parameters (the control tests and their results are detailed in the Supplementary material 1). First, all other parameters being equal, the amount of time spent by gammarids in the laboratory had no influence on infection dynamics, in terms of development time of parasites, infection success, parasite load and behavioral manipulation. Only activity was reduced in gammarids that spent more time under laboratory conditions. Second, gammarids tested at the same absolute parasite age (in days) showed differences in their behavior consistent with the idea that manipulation is linked to the parasite stage^73^, and not to the time that parasites did spend in gammarids. Indeed, gammarids infected with acanthella stages behaved significantly differently than gammarids infected with cystacanth stages of the same age.

In addition to the parasite’s development time, temperature also modified other infection parameters in our study. When exposure to parasite eggs occurred at different temperatures with hosts previously acclimatized at these temperatures, infection success was slightly higher at 17 °C compared to 14 °C and more parasites per host developed at 17 °C compared to 14 °C in these conditions, although these two differences were only marginally significant. These results agree with those found in *Squalius cephalus*, a definitive fish host of *P. laevis* in which a higher probability of infection and a higher parasite load was found at 22 °C compared to 18 °C^60^. The fact that a difference was observed only when exposure to parasite eggs occurred at different temperatures suggests that the effect of temperature on these two parameters was due to a higher consumption of eggs rather than a higher success of establishment of the parasites in their hosts. This hypothesis is supported by several studies that have reported a positive effect of temperature on food consumption by gammarids^23,87,89^, which may have affected their probability of consuming parasite eggs in the present study.

The latency of parasite to evert their proboscis, a parameter used as a proxy for parasite stamina^85^, was also affected by temperature, with parasites that developed at 17 °C starting the eversion sooner after the adding of fish bile compared to those which developed at 14 °C. During this test, the temperature was similar between the two groups, such that only the temperature experienced during parasite development could affect their proboscis eversion. This result, along with the faster development of parasites inside their hosts, may stem from an increased metabolism of parasites at 17 °C compared to 14 °C. The observed negative correlation between the rapidity of proboscis eversion and the magnitude of manipulation in infected gammarids from the first exposure suggests that the two phenomena might not be independent. However, the correlation was very weak and significant only at 17 °C for the third behavior round. Such negative correlation, if confirmed, could underlie a trade-off between parasite stamina and manipulation. Similarly, Maure et al.^90^ found a negative correlation between the fecundity of the parasitic wasp *Dinocampus coccinellae* and the length of the period of manipulation of its beetle host. Such correlations were not found in other studies^45,70^, although not directly tested, and the existence of such trade-offs thus needs to be further investigated.

In parallel to its effect on parasite traits, temperature also affected certain traits of the hosts. First, the survival of gammarids was decreased at high temperature, as already shown in other studies^55,56^, while infection with parasites also led to a higher mortality of gammarids. However, the influence of parasites on host survival was not affected by temperature.

Activity level, a parameter tightly linked to metabolism in gammarids^54^, was also affected by temperature. As expected, gammarids were globally more active at high temperature^54,91^. This difference could however be partly linked to the fact that gammarids kept at low temperature were tested after a longer time in laboratory conditions, as the second part of the experiment showed that a longer maintenance led to a decrease in the activity level of gammarids. In parallel, infection with acanthocephalan parasites has been shown to increase the activity of gammarids compared to that of uninfected ones in several studies^92–94^, although contradictory results were also reported^72,95,96^. Interestingly, our results suggest that abiotic conditions could be responsible for such contradictions. Indeed, infected individuals were significantly more active than control individuals only at 14 °C. On the contrary, although this difference was never significant, average activity level was slightly higher in control individuals compared to infected ones at 17 °C, in all our experimental infections.

Altogether, these results suggest that the metabolism of both hosts and parasites was accelerated at 17 °C compared to 14 °C. In contrast, no difference was observed according to temperature in the behavior of gammarids in terms of refuge use, neither in the timing of manipulation nor in its intensity. As already stated before, refuge use tended to increase with time for control individuals, while infected individuals decreased their use of refuges through time^45,97^. These two trends were observed at both temperatures, with the same progressive manipulation of infected individuals. Only the number of parasites per hosts was found to influence the use of refuges. Manipulation was delayed in gammarids harboring more than two parasites. However, this phenomenon might result from our protocol, that could not control for competition between parasites of different stages within the same host. Indeed, the behavior of individuals was tested as soon as one cystacanth was detected through gammarids cuticle. Variation in the growth of parasites sharing the same host has been shown^70^, and a small asynchrony is thus expected to occur in the exact day of switching to the cystacanth stage in gammarids with multiple acanthocephalan infection. It is thus likely that multi-infected gammarids still harbored acanthella parasites when they were first tested, or parasites at an earlier cystacanth stage in the second test. The manipulation of gammarids by parasites at the cystacanth stage is known to be reduced by the presence of acanthella parasites within the host^97^, a stage known to enhance the anti-predatory behaviors of their hosts rather than reducing it^49^, thus potentially explaining the delayed manipulation observed in our study.

The absence of any effect of temperature on manipulation, in terms of use of refuges, suggests that manipulation, at least on this dimension, might not be plastic, contrary to what has been previously proposed^45^. Indeed, as the survival of gammarids decreased at high temperature, we would expect ideally adapted parasites to adopt a strategy to increase their chances of being transmitted before the death of their hosts, such as a faster manipulation^98^. Interestingly, in a similar study investigating the effect of host nutritional condition, Labaude et al.^45^ also found that, although the survival of gammarids was reduced by a poor diet, along with effects on other metabolic traits, the amount of host resources had no effect on *P. laevis* manipulation of *G. pulex*.

Nevertheless, temperature might have affected parasite manipulation in a way that could not be detected in our study. First, tests occurred in the absence of any clue from predators. Although such conditions are sufficient to induce alterations in behavior, the differences between control and infected individuals might have been exacerbated by the presence of a predator odour^38,47,99–101^. Second, variations induced by temperature might be associated to other behaviors than the use of refuges. Indeed, the seasonal variation of manipulation observed by Franceschi et al.^71^ in gammarids infected with acanthocephalans was evidenced while assessing phototaxis, whereas the use of refuges was not tested. A recent study investigating the effects of a shorter acclimatization time at different temperatures on control and gammarids naturally infected by *P. tereticollis* showed a significant effect of temperature on the phototaxis of both infected and control gammarids, but not on their use of refuges^22^. By contrast, Benesh et al.^67^ showed that different experimental conditions of light and temperature, chosen to mimic seasonal differences, altered the use of refuges of both infected and uninfected isopods. However, the difference of behavior between infected and uninfected individuals remained similar under each experimental condition, although the two parameters were not investigated separately. On the contrary, they found that this difference varied among isopods collected at different seasons. A seasonal effect of manipulation was also documented in gammarids infected by acanthocephalan parasites^71^, although the mechanisms explaining such seasonality were not identified. Our study supports the hypothesis made by Benesh et al.^67^ who suggested that seasonal changes in manipulation might not be caused by proximal abiotic conditions. In other studies, only light properties were shown to affect manipulation^68,69^, while other factors such as the quantity of resources available did not affect manipulation either^45^. Thus, seasonality in manipulation could depend upon other parameters. We might expect such variation to rely on an internal clock with a genetic basis, although the mechanisms responsible for such timing need to be investigated. In this case, global change might alter the seasonal distribution and/or the diet of definitive fish hosts and ultimately lead to a maladaptation of the degree of manipulative efforts of parasites.

Although temperature did not plastically affect the manipulation of acanthocephalan parasites in our study, indirect effects are likely to occur^102^. Indeed, temperature was shown to be linked to leaf consumption by gammarids^23,87,89^, ultimately leading to higher infection success and parasite load. Such an effect was also observed in the definitive hosts of parasites^60^. In addition, parasites developed faster at high temperature. Other conditions, such as the availability of resources known to modulate parasite load in their gammarid hosts^45^, are also likely to be affected by temperature. Altogether, these effects might lead to modifications of the intensity of infection, known to influence manipulation^73^, as well as in the prevalence of acanthocephalan parasites in gammarid populations, thus, provided that prevalence is high enough, modifying behaviors at the population level.

To our knowledge, this study is the first to investigate the effect of temperature on the timing and intensity of behavioral changes using experimental infections. Our results provide solid evidence that temperature might affect many parameters of host–parasite associations, with no direct effect on the extent of certain important traits of behavioral manipulation. In addition to an increase of temperature as tested here, climate changes might also lead to an increase in the frequency of extreme climatic events, leading to more fluctuations in temperature regimes^103^. In particular, the effect of sudden increases of temperature at a higher temperature than the one tested in this study would be interesting to investigate. Finally, although temperature might not be directly responsible for changes in the behavior of gammarids, further studies are needed to investigate the generality of its effect in other host–parasite associations, before concluding about how its interaction with manipulative parasites might alter the functional role of gammarids within food webs. To that end, it would be worth considering other development stages of parasites, as well as the behavior of the definitive host, in more integrative experiments. The relevance of studies focusing on the impact of global changes on manipulative parasites has already been suggested^102^, and other host–parasite systems should also be investigated.

## Supplementary information

Supplementary information.

## Data Availability

The datasets generated during and/or analyzed during the current study are available from the corresponding author on reasonable request.
