# Supplementary material for: Increased temperature has no consequence for behavioral manipulation despite effects on both partners in the interaction between a crustacean host and a manipulative parasite
Source: Sci Rep. 2020 Jul 15;10:11670. doi: 10.1038/s41598-020-68577-z (PMC7363812; doi:10.1038/s41598-020-68577-z)
Supplement: Supplementary file 1 — Supplementary information. [file 41598_2020_68577_MOESM1_ESM.docx]

**SUPPLEMENTARY MATERIAL**

**Increased temperature has no consequence for behavioral manipulation despite effects on both partners in the interaction between a crustacean host and a manipulative parasite**

Sophie Labaude, Frank Cézilly, Lila De Marco & Thierry Rigaud

1. **Effect of the absolute time spent by parasites inside their hosts**
   1. Methods

The behaviour of 35 gammarids exposed to parasites and maintained at 14°C (thus expected to harbour parasites at the acanthella stage, as was subsequently confirmed on dissection) was tested at the same time as the behaviour of gammarids maintained at 17°C (Fig. 1). Control individuals, maintained at 14°C, were tested in parallel. The behavioural scores of infected gammarids kept at 14°C (that were still at the acanthella stage) were compared with those of individuals kept at 17°C (that had already reached the cystacanth stage), using Wilcoxon tests and post hoc comparisons (Fig. 1).

- 1. Results

Refuge use differed between individuals tested simultaneously, according to temperature and infection status (Wilcoxon, *χ*^2^ = 80.96, d.f. = 3, *P* < 0.0001). Post hoc comparisons showed that the scores of individuals harbouring cystacanth stages (17°C) were significantly higher than those of both individuals infected with acanthella (14°C) and control individuals (14°C and 17°C; Fig. S1A). The scores of individuals infected with acanthella did not differ from that of control individuals (Fig. S1A). The activity of gammarids was significantly affected by temperature (*F*_245, 1_ = 30.45, *P* < 0.0001), but not by infection status. Tukey post-hoc tests showed that activity was higher at 17°C compared to 14°C, for all infection status (Fig. S1B).


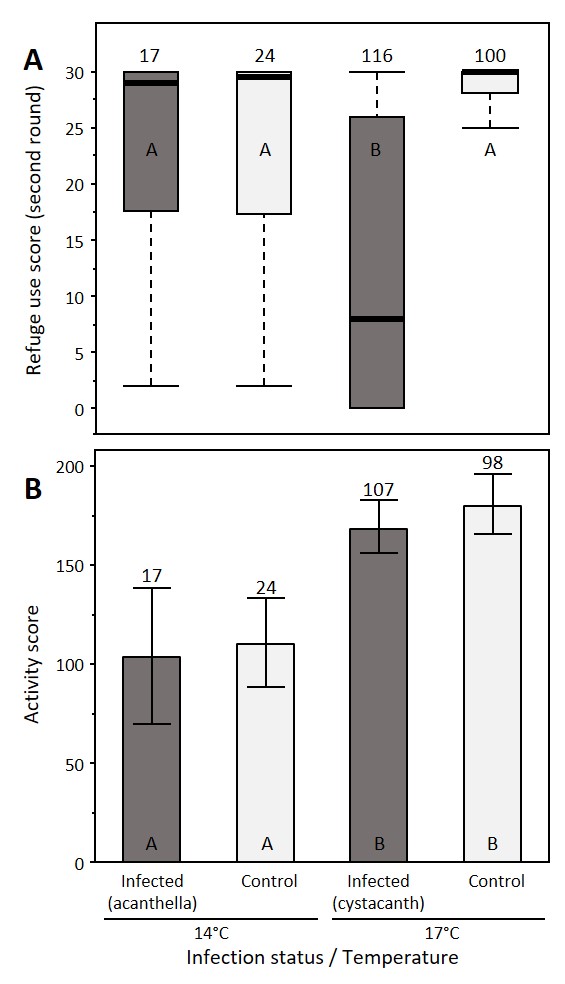


**Fig. S1.** Behavioural scores according to temperature (14°C and 17°C) and infection status (infected and control). (A) Score for refuge use (median, upper and lower quartiles and deciles) measured during the second round (eight days after the detection of cystacanth stages at 17°C) and (B) activity level of gammarids (mean and 95% confidence intervals). All individuals were tested simultaneously, such that infected gammarids at 17°C harboured parasites at the cystacanth stage while parasites were still at the acanthella stage in gammarids maintained at 14°C. Sample sizes are given above each bar. Significant differences are indicated by different letters (Tukey’s HSD post hoc tests; *P* < 0.05).

1. **Effect of the time spent by gammarids in the laboratory (17°C)**
2. Methods

The effect of the time spent by gammarids in the laboratory, which also differed between the two temperature treatments (14°C and 17°C), was controlled by comparing the behavioural scores of individuals from the second exposure that spent long vs. short time in the laboratory before their infection (respectively first sampling, in October, vs. second sampling, in November).

1. Results

No differences were observed in infection parameters between gammarids maintained for short time vs. long time in the laboratory (development time: Mann-Whitney U test, *Z* = 0.61, *P* = 0.54; infection success: LR-*χ*^2^ = 0.36, d.f. = 1, P = 0.55; parasite load: Mann-Whitney U test, *Z* = -0.22, *P* = 0.82). In addition, refuge use was not affected by the time spent by gammarids in the laboratory. After removing this factor from the analysis, factors found to influence the use of refuges remained the same (status: Statistic = 29.11, d.f. = 1, *P* < 0.0001; round: Statistic = 45.46, d.f. = 1.92, *P* < 0.0001; and their interaction: Statistic = 44.96, d.f. = 1.92, *P* < 0.0001). There was no effect of time of maintenance on latency time before proboscis eversion, and this parameter was thus removed from the Generalized Linear Model. The remaining model (*χ*^2^ = 13.18, d.f. = 2, *P* = 0.0014) showed that the time needed by parasites to start everting their proboscis was negatively and significantly influenced by the size of their hosts (*χ*^2^ = 6.86, d.f. = 1, *P* = 0.0088) and by parasite load (*χ*^2^ = 6.53, d.f. = 1, *P* = 0.011), while these two parameters were independent (Spearman’s rho = -0.026, n = 58, *P* = 0.85). Time in laboratory only affected activity level, with lower score for gammarids maintained for the longer amount of time in the laboratory (*F*_1, 59_ = 4.63, *P* = 0.036), whereas the effect of infection status was not significant (Fig. S2).


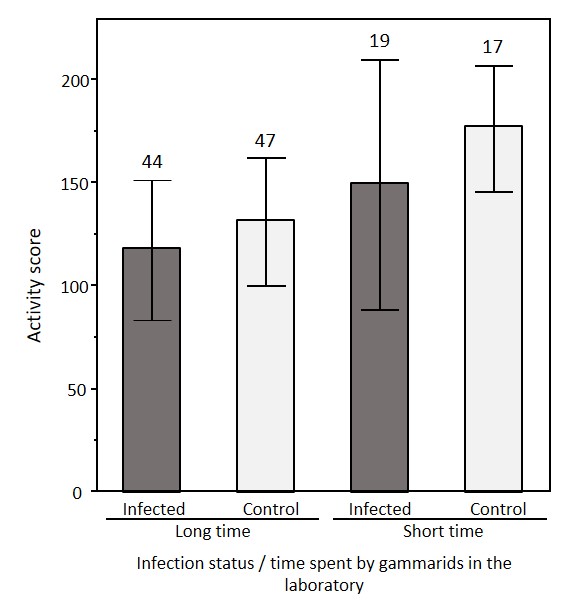


**Fig. S2.** Scores of the activity level of gammarids according to infection status (infected and control) and time spent in the laboratory before being infected (long time vs short time, respectively first sampling and second sampling of gammarids). Sample sizes are given above each bar. Mean values and 95% confidence intervals are indicated.
